# Relationship of the Perceived Social and Physical Environment with Mental Health-Related Quality of Life in Middle-Aged and Older Adults: Mediating Effects of Physical Activity

**DOI:** 10.1371/journal.pone.0120475

**Published:** 2015-03-23

**Authors:** Delfien Van Dyck, Megan Teychenne, Sarah A. McNaughton, Ilse De Bourdeaudhuij, Jo Salmon

**Affiliations:** 1 Research Foundation Flanders, Egmontstraat 5, 1000 Brussels, Belgium; 2 Ghent University, Department of Movement and Sports Sciences, Watersportlaan 2, 90000 Ghent, Belgium; 3 Deakin University, Centre for Physical Activity and Nutrition Research, School of Exercise and Nutrition Sciences, 221 Burwood Highway, Burwood, Victoria 3125, Australia; Cinvestav-Merida, MEXICO

## Abstract

**Background:**

Mental health conditions are among the leading non-fatal diseases in middle-aged and older adults in Australia. Proximal and distal social environmental factors and physical environmental factors have been associated with mental health, but the underlying mechanisms explaining these associations remain unclear. The study objective was to examine the contribution of different types of physical activity in mediating the relationship of social and physical environmental factors with mental health-related quality of life in middle-aged and older adults.

**Methods:**

Baseline data from the Wellbeing, Eating and Exercise for a Long Life (WELL) study were used. WELL is a prospective cohort study, conducted in Victoria, Australia. Baseline data collection took place in 2010. In total, 3,965 middle-aged and older adults (55–65 years, 47.4% males) completed the SF-36 Health Survey, the International Physical Activity Questionnaire, and a questionnaire on socio-demographic, social and physical environmental attributes. Mediation analyses were conducted using the MacKinnon product-of-coefficients test.

**Results:**

Personal safety, the neighbourhood physical activity environment, social support for physical activity from family or friends, and neighbourhood social cohesion were positively associated with mental health-related quality of life. Active transportation and leisure-time physical activity mediated 32.9% of the association between social support for physical activity from family or friends and mental health-related quality of life. These physical activity behaviours also mediated 11.0%, 3.4% and 2.3% respectively, of the relationship between the neighbourhood physical activity environment, personal safety and neighbourhood social cohesion and mental health-related quality of life.

**Conclusions:**

If these results are replicated in future longitudinal studies, tailored interventions to improve mental health-related quality of life in middle-aged and older adults should use a combined strategy, focusing on increasing physical activity as well as social and physical environmental attributes.

## Introduction

Mental health conditions including depression and anxiety, are among the leading non-fatal diseases in Australia and are associated with high direct and indirect health care costs, and increased risk of mortality from cardio-vascular disease and other chronic diseases, as well as suicide [1–4]. Research from Australia has shown that 8–10% of the population suffers from depression and that 14–20% suffers from anxiety [5]. In order to prevent and treat these and other mental health problems, it is important to understand the full range of determinants that may contribute to the development of mental health conditions.

Most previous research has focused on the individual and proximal (i.e. close to the individual) social determinants of mental health. Consistent evidence shows that the prevalence of mental health conditions (e.g. depression and anxiety) is higher in women than in men as well as being higher in adults who are single or in a poor-quality relationship compared to those who are in a good-quality relationship [5–7]. Furthermore, the prevalence of mental health conditions is higher in adults of low socio-economic position [8] and decreases with increasing age [9,10], although depression in particular remains a highly prevalent mental health condition in older adults [11]. Longitudinal studies showed that social support from family/friends and interactions with family and friends are two proximal social factors that influence mental health in (older) adults [12,13].

Recently, increasing attention has been paid to the importance of the distal (i.e. further away from the individual) social and physical environment in explaining mental health. Research has shown that the neighbourhood environment is a setting which exposes people to factors that can be either beneficial or harmful to mental health [14]. Having positive perceptions of characteristics relating to neighbourhood social capital (neighbourhood social cohesion, interpersonal trust and norms of reciprocity) has been consistently related to a lower prevalence of mental health conditions in both cross-sectional and longitudinal studies conducted in the general population [15–18] and in older adults [19]. Neighbourhood physical environmental factors have been studied less often in relation to mental health, but some evidence exists that perceptions of high safety, high walkability, good access to parks and favourable neighbourhood aesthetics are associated with better overall mental health compared to less positive environmental attributes [20–23]. Although most results on this topic are from cross-sectional studies, some longitudinal evidence supports a causal relationship of perceived safety and residential density with mental health [18,24].

However, the mechanisms which explain the associations between proximal and distal social and physical environmental factors with mental health remain unclear. Previous studies reported on the mediating effects of psychological distress, sense of control and friendship quality to explain the relationship of neighbourhood social capital and perceived safety with mental health [14,23]. Nonetheless, physical activity may also be an important mediator. The Social Interaction Theory of Ransford [25] states that social relationships can improve mental health through physical activity. Furthermore, based on existing empirical evidence, Diez Roux & Mair [26] proposed a conceptual framework suggesting that behavioural factors like physical activity and eating habits may be important mediators in the relationship of neighbourhood physical and social environments with both mental and physical health.

Next to these theoretical assumptions, empirical evidence from cross-sectional and longitudinal studies shows that proximal and distal psychological and social environmental factors (e.g. social support for physical activity, self-efficacy, neighbourhood social cohesion), as well as neighbourhood physical environmental factors (e.g. walkability, safety, aesthetics, proximity to recreation facilities) are related to physical activity [27–31]. In turn, increased levels of physical activity lead to improved mental health [9,32,33]. This existing evidence concerning both relationships (environment—physical activity and physical activity—mental health) as well as the proposed theoretical frameworks, suggest that a mediating effect of physical activity may be present. However, to our knowledge no previous studies have examined this mediating role of physical activity.

**Fig 1:**
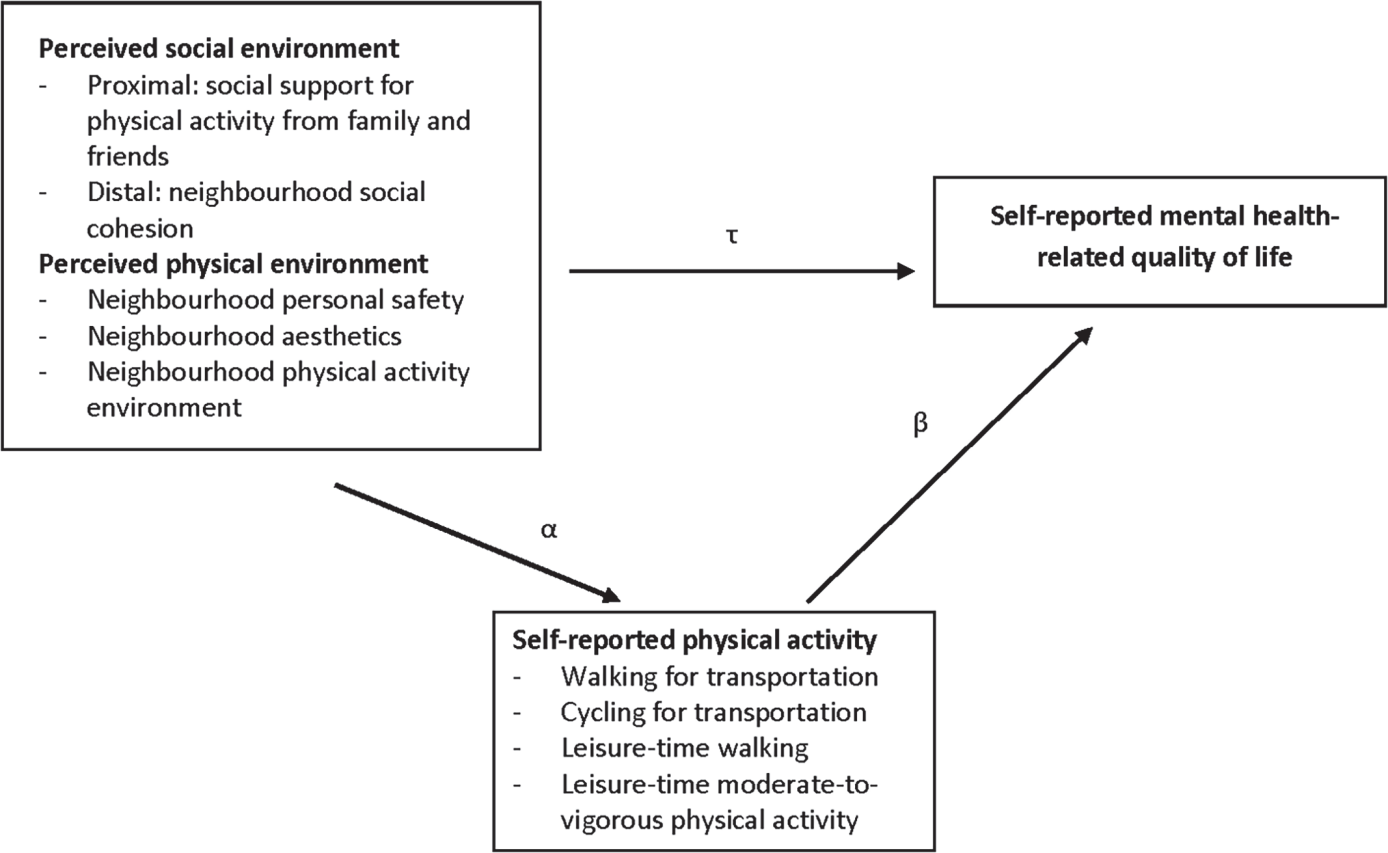
Conceptual model of pathways and variables examined through the mediation analyses. τ = main associations between the physical/social environmental perceptions and mental health-related quality of life. α = associations between the physical/social environmental perceptions and potential mediators. β = independent associations between potential mediators and mental health-related quality of life. Educational level, employment status, smoking status, marital status, BMI, and work- and household-related physical activity were included as covariates in all analyses.

In order to shed light on the gaps in the current literature, the present study was conducted. The aim of this study was to examine the contribution of different types of physical activity in mediating the relationship of social (proximal and distal) and physical environmental factors with mental health (assessed as mental health-related quality of life) in middle-aged and older adults. Fig. 1 clarifies the pathways and variables that were examined in this study. Since it has been shown that active transportation (walking and cycling) and leisure-time physical activity (walking and moderate-to-vigorous physical activity [MVPA]) in particular may be beneficial for mental health (mainly evidence for prevention of depression) [33–35], we have focused upon these types of physical activity. In addition, household- and work-related activities were also assessed to obtain information on a full day’s physical activity. It is especially important to study these relationships in middle-aged and older adults since this age group is rising rapidly in developed countries [36]. Further, neighbourhood social and physical environmental factors are potentially more important for older adults than for younger adults, since older adults are typically more attached to their neighbourhood and perform most of their activities there [14].

## Materials and Methods

This study used data from the Wellbeing, Eating and Exercise for a Long Life (WELL) study. Details on the study protocol have been published elsewhere [37]. Briefly, the WELL study was a prospective cohort study of middle-aged and older adults (55–65 years at baseline) examining nutrition, physical activity behaviours, risk of overweight/obesity, quality of life and their influencing factors. The study was conducted in Victoria, Australia. The state of Victoria is located in the south-east of Australia, covers an area of 227.4 km^2^, has a population of approximately 5,713,000, 49% males, and a population density of 23.9 persons/km^2^. In 2009, 11% of the inhabitants was aged between 55 and 65 years [38]. Baseline data collection took place in 2010.

### Ethics Statement

The study was approved by the Deakin University Human Research Ethics Committee (2009–105) and all participants provided written informed consent.

### Procedure and participants

Participants were selected from the Australian Electoral Commission (AEC) using a stratified random sampling process. In a first step, suburbs in Victoria were classified as urban or rural, and suburbs with populations of less than 1000 or less than 200 55–65 year olds were excluded. Second, all remaining suburbs were classified by socioeconomic position according to the socioeconomic Index for Areas score (SEIFA) [39] and divided into tertiles (low, medium, high). From each SEIFA tertile, 14 postcodes were randomly selected: seven in urban areas and seven in rural areas. Furthermore, an equal number of men and women from urban and rural suburbs in each tertile were randomly selected. This selection procedure resulted in a total sampling pool of 11,256 possible participants.

Potential participants were sent an invitation letter to participate in the study. One week later, the survey was sent with a reply-paid envelope for survey return. Up to two reminder letters (three weeks and six weeks after mailing the survey) were sent to those who did not return their survey [40]. Of the surveys distributed, 380 were returned as undeliverable and 95 were returned from individuals outside the 55–65 age range. In total, 4,082 completed surveys were returned (38% response rate). This response rate is similar to what is usually achieved by postal questionnaires of this kind [41]. As physical activity behaviours were examined as potential mediators in the present study, only those participants who reported that their health did not prevent them from walking one hundred metres and/or bathing or dressing themselves, were included in the analyses. Applying this exclusion criterion lead to a final sample of 3,965 participants. Socio-demographic characteristics of the sample are shown in Table 1.

**Table 1:** Socio-demographic characteristics and average scores on dependent, explanatory and mediating variables.

Variable	Total sample(n = 3965)
**Socio-demographic characteristics**	
Gender (%)	
Men	47.4
Women	52.6
Age (mean [SD]	60.3 (3.2)
Educational level (%)	
Low	36.7
Medium	35.9
High	27.4
Employment status (%)	
Employed	59.4
Unemployed/retired	40.6
Smoking status (%)	
Smoker	12.1
Non-smoker	87.9
Marital status (%)	
Married/in a relationship	77.9
Alone	22.1
Residential location (%)	
Urban	46.8
Rural	53.2
Body Mass Index (mean [SD])	27.1 (4.7)
**Physical activity behaviour (mean [SD])**	
Walking for transportation (min/week)	186.3 (260.3)
Cycling for transportation (min/week)	13.6 (64.7)
Leisure-time walking (min/week)	142.9 (208.0)
Leisure-time moderate-to-vigorous physical activity (min/week)	84.0 (176.5)
Work-related total physical activity	402.4 (578.7)
Household-related total physical activity	628.9 (554.7)
**Physical environmental perceptions (mean [SD])** ^**a**^	
Neighbourhood personal safety, mean of 3 items	3.6 (0.8)
Neighbourhood aesthetics, mean of 5 items	3.8 (0.5)
Neighbourhood physical activity environment, mean of 7 items	3.9 (0.6)
**Social environmental perceptions (mean [SD])**	
Social support for physical activity from family or friends ^b^	2.6 (1.1)
Social cohesion of the neighbourhood ^a^	3.5 (0.6)
**Overall mental health-related quality of life (mean [SD])** ^**c**^	79.2 (18.0)

SD = standard deviation.

^a^ positively scored on a 5-point Likert scale (1–5), ranging from ‘strongly disagree’ to ‘strongly agree’.

^b^ positively scored on a 5-point Likert scale (1–5), ranging from ‘never’ to ‘very often’.

^c^ positively scored on a scale from 0–100; higher score = better overall mental health-related quality of life.

### Measures

#### Outcome variable: Mental health-related quality of life

Mental health-related quality of life was assessed as a proxy of self-reported mental health. This was done using a validated version of the SF-36 Health Survey [42]. This questionnaire contains 36 items that measure eight multi-item dimensions of health status. Four of these dimensions relate to overall mental health status during the past four weeks: vitality (4 items), social functioning (2 items), role limitations due to emotional problems (role emotional; 3 items) and mental health components referring to different states of mind (5 items). To calculate each dimension, item scores were coded, summed and transformed to a scale from 0 to 100, with higher scores indicating better mental health. Finally, a mean score of the four dimensions was calculated to obtain an overall standardized mental health-related quality of life summary score, ranging from 0 to 100. The four dimensions of the SF-36 Health Survey relating to overall physical health status during the past four weeks were not used in the present study.

#### Explanatory variable: Perceived neighbourhood physical environment

Existing measures were used to assess perceptions of neighbourhood personal safety (mean of three items, Cronbach’s alpha = 0.79), neighbourhood aesthetics (mean of five items, Cronbach’s alpha = 0.69) and the neighbourhood physical activity environment (mean of seven items, Cronbach’s alpha = 0.84) [43]. All items were assessed using a five-point Likert scale, ranging from ‘strongly disagree’ (1) to ‘strongly agree’ (5).

For neighbourhood personal safety, agreement with the following statements was assessed: ‘I feel safe walking in my neighbourhood, day or night’, ‘Violence is not a problem in my neighbourhood’, and ‘My neighbourhood is safe from crime’. For neighbourhood aesthetics, agreement with the following five statements was assessed: ‘There is a lot of rubbish on the street in my neighbourhood’, ‘There is a lot of noise in my neighbourhood’, ‘In my neighbourhood the buildings and homes are well-maintained’, ‘The buildings and homes in my neighbourhood are interesting’, and ‘My neighbourhood is attractive’. For neighbourhood physical activity environment, agreement with the following seven statements was assessed: ‘My neighbourhood offers many opportunities to be physically active’, ‘Local sports clubs and other facilities in my neighbourhood offer many opportunities to get exercise’, ‘It is pleasant to walk in my neighbourhood’, ‘The trees in my neighbourhood provide enough shade’, ‘In my neighbourhood it is easy to walk places’, ‘I often see other people walking in my neighbourhood’, and ‘I often see other people exercising in my neighbourhood’.

#### Explanatory variable: Perceived proximal and distal social environment

Two perceived social environment constructs were assessed: social support for physical activity from family or friends (proximal social environment; mean of four items, Cronbach’s alpha = 0.71) and social cohesion of the neighbourhood (distal social environment; mean of five items, Cronbach’s alpha = 0.80) [44].

Social support for physical activity from family/friends was assessed by asking how often family/friends offered to do physical activity with the participant and how often family/friends encouraged the participant to be physically active. These items were assessed using a five-point Likert scale, ranging from ‘never’ (1) to ‘very often’ (5). For social cohesion of the neighbourhood, agreement with the following five items was assessed: ‘People in my neighbourhood can be trusted’, ‘This is a close-knit neighbourhood’, ‘People around here are willing to help their neighbours’, ‘People in this neighbourhood generally do not get along with each other’ and ‘People in the neighbourhood do not share the same values’. Items were assessed with a five-point Likert scale ranging from ‘strongly disagree’ (1) to ‘strongly agree’ (5).

#### Mediators: Self-reported active transportation and leisure-time physical activities

Self-reported physical activity was collected using the long version of the International Physical Activity Questionnaire (IPAQ-L). The IPAQ is suitable for use in adults aged 15–69 years, has excellent test-retest reliability properties (pooled r = 0.81) and acceptable validity (pooled r = 0.33) when compared with accelerometer-based physical activity [45]. Frequency (number of days in the last seven days) and duration (hours and minutes per day) of physical activity in different domains (work, transportation, recreation and household) were assessed. Minutes/week of walking for transportation, cycling for transportation, leisure-time walking and leisure-time MVPA were calculated and included in the analyses as potential mediators. To allow other forms of physical activity to be controlled for, weekly minutes of work-related (walking and MVPA) and household-related (MVPA in home and garden) were calculated as well.

#### Covariates

Possible covariates were selected based on evidence from previous literature examining associations of socio-demographic characteristics with depression, anxiety and health-related quality of life [5,7–9,46]. Self-reported information on participants’ sex, age, education level (low: <Year 12; medium: Year 12/trade/certificate; high: university/postgraduate), employment status (employed, including full-time work and part-time work; not employed, including those unemployed/laid off, keeping house/raising children, full time study and retired), smoking status (yes; no, including never and former), marital status (married/living as married; alone, including separated, divorced, widowed and never married), residential location (urban; rural) and body mass index (BMI; self-reported weight in kilograms divided by height in metres squared) were collected. The variables that correlated significantly with mental health-related quality of life were included as covariates in the analyses. Consequently, educational level, employment status, smoking status, marital status and BMI were included as covariates in all analyses, as well as work- and household-related physical activity.

### Statistical analyses

Analyses were conducted using SPSS 19.0. As the physical activity variables were positively skewed, a logarithmically transformed variable (log10) was used in the analyses to improve normality [47]. Raw data were used to calculate the descriptive statistics, shown in Table 1. In all analyses, clustering of participants at area level was taken into account by conducting multilevel analyses (two-level: level 1 = individual; level 2 = area of residence). A matrix representing Pearson correlations between all explanatory variables, mediators and the outcome variable is provided as a supplementary file (S1 Table). In the first stage of the analyses, the main associations between the physical/social environmental perceptions and mental health-related quality of life were tested (τ), using multiple linear mixed models analyses, with mental health-related quality of life as the dependent variable. The different physical/social environmental perceptions were independent variables. In case of a significant association between one of the physical/social environmental perceptions and mental health-related quality of life, the second stage of the analyses was conducted for that particular variable. In that second stage, the mediating role of different types of physical activity was tested using the product-of-coefficients test of MacKinnon et al [48]. This specific test was used because it provides greater statistical power than other commonly used mediating methods [49]. The test includes (1) estimating the effects of the physical/social environmental perceptions on potential mediators (transport-related walking and cycling, leisure-time walking, leisure-time MVPA; α-coefficients) by regressing the potential mediators onto the physical/social environmental perceptions; (2) estimating the independent effect of the potential mediators on mental health by regressing mental health-related quality of life onto the physical/social environmental perceptions and the potential mediators (β-coefficients); (3) computation of the product of the two coefficients (αβ), representing the mediated effect. The statistical significance of the mediated effect was estimated by dividing the product-of-coefficient by its standard error. Moreover, the proportion mediated was calculated by dividing the product-of-coefficient (αβ) by the total main effect of the physical/environmental perceptions on mental health-related quality of life (τ; first stage of analyses). For all analyses, 95% confidence intervals (CI) were reported.

## Results

### Main associations between physical/social environmental perceptions and mental health-related quality of life (τ; Table 2)

The results of the multiple linear mixed model analyses showed that neighbourhood personal safety (CI = 1.54, 3.22), neighbourhood physical activity environment (CI = 1.82, 4.11), social support for physical activity from family or friends (CI = 0.67, 1.75) and social cohesion of the neighbourhood (CI = 1.59, 3.81) were positively associated with mental health-related quality of life. Perceived neighbourhood aesthetics (CI = -0.96, 1.55) was not related to mental health-related quality of life. Based on these findings, the potential mediators of the significant associations of neighbourhood personal safety, neighbourhood physical activity environment, social support for physical activity from family or friends and the social cohesion of the neighbourhood with mental health-related quality of life were examined.

**Table 2:** Main effects (τ [SE])of physical and social environmental perceptions on mental health-related quality of life.

Independent variables	τ (SE)	95% CI for τ
Neighbourhood personal safety	2.38 (0.43)	1.54, 3.22
Neighbourhood aesthetics	0.29 (0.64)	−0.96, 1.55
Neighbourhood physical activity environment	2.97 (0.59)	1.82, 4.11
Social support for PA from family or friends	1.21 (0.28)	0.67, 1.75
Social cohesion of neighbourhood	2.70 (0.57)	1.59, 3.81

SE = standard error; CI = confidence interval; PA = physical activity.

*Note 1*: the analysis was adjusted for individual-level BMI, marital status, smoking status, educational level and employment status, work-related and household-related total physical activity.

*Note 2*: mental health was the dependent variable in all analyses.

### Associations between physical/social environmental perceptions and potential mediators (α-coefficients; Table 3)

Neighbourhood personal safety (model 1) was positively associated with transport-related cycling (CI = 0.02, 0.10) and with leisure-time MVPA (CI = 0.01, 0.14), but not with the other potential mediators. Perceiving a better neighbourhood physical activity environment (model 2) was associated with more transport-related walking (CI = 0.15, 0.34), more transport-related cycling (CI = 0.04, 0.15), more leisure-time walking (CI = 0.29, 0.47) and more leisure-time MVPA (CI = 0.19, 0.37). Social support for physical activity from family or friends (model 3) was positively associated with transport-related walking (CI = 0.14, 0.23), transport-related cycling (CI = 0.03, 0.08), leisure-time walking (CI = 0.26, 0.36) and leisure-time MVPA (CI = 0.27, 0.37). Finally, perceiving higher levels of social cohesion of the neighbourhood (model 4) was positively associated with leisure-time walking (CI = 0.01, 0.19) and leisure-time MVPA (CI = 0.08, 0.26). Only for those potential mediators that were significantly related with the physical/social environmental perceptions, β-coefficients were calculated.

**Table 3:** Regression analyses for possible mediators of the associations between physical/social environmental perceptions and mental health-related quality of life.

Possible mediators	α (SE)	95% CI for α	β (SE)	95% CI for β	αβ (SE)	95% CI for αβ	Proportion mediated (%)
*Model 1: Neighbourhood personal safety*							
Walking transport	0.05 (0.04)	−0.02, 0.12					
Cycling transport	0.06 (0.02)	0.02, 0.10	0.96 (0.33)	0.31, 1.61	0.06 (0.03)	0.01, 0.11	**1.5**
Leisure-time walking	0.07 (0.04)	−0.01, 0.14					
Leisure-time MVPA	0.07 (0.03)	0.01, 0.14	1.07 (0.19)	0.70, 1.45	0.08 (0.04)	0.01, 0.14	**1.9**
**Total multiple model**					0.09 (0.04)	0.01, 0.17	**2.3**
*Model 2: Neighbourhood physical activity environment*							
Walking transport	0.25 (0.05)	0.15, 0.34	0.45 (0.19)	0.08, 0.83	0.11 (0.05)	0.01, 0.22	**2.1**
Cycling transport	0.09 (0.03)	0.04, 0.15	0.95 (0.33)	0.30, 1.59	0.09 (0.04)	0.01, 0.17	**1.6**
Leisure-time walking	0.38 (0.05)	0.29, 0.47	0.85 (0.19)	0.48, 1.22	0.32 (0.08)	0.16, 0.49	**6.1**
Leisure-time MVPA	0.28 (0.05)	0.19, 0.37	0.93 (0.19)	0.56, 1.30	0.26 (0.07)	0.12, 0.40	**4.9**
**Total multiple model**					0.58 (0.10)	0.38, 0.78	**11.0**
*Model 3: Social support for physical activity from family or friends*							
Walking transport	0.19 (0.02)	0.14, 0.23	0.52 (0.20)	0.14, 0.90	0.10 (0.04)	0.02, 0.18	**5.6**
Cycling transport	0.06 (0.02)	0.03, 0.08	1.08 (0.33)	0.44, 1.74	0.07 (0.03)	0.01, 0.12	**3.7**
Leisure-time walking	0.31 (0.02)	0.26, 0.36	0.90 (0.19)	0.52, 1.28	0.28 (0.06)	0.16, 0.40	**15.9**
Leisure-time MVPA	0.32 (0.02)	0.27, 0.37	0.97 (0.19)	0.59, 1.36	0.31 (0.06)	0.19, 0.44	**17.6**
**Total multiple model**					0.58 (0.08)	0.42, 0.74	**32.9**
*Model 4: Social cohesion neighbourhood*							
Walking transport	0.07 (0.04)	−0.02, 0.16					
Cycling transport	0.01 (0.03)	−0.04, 0.06					
Leisure-time walking	0.10 (0.05)	0.01, 0.19	1.02 (0.19)	0.66, 1.39	0.10 (0.05)	−0.01, 0.21	
Leisure-time MVPA	0.17 (0.04)	0.08, 0.26	1.04 (0.19)	0.67, 1.41	0.18 (0.05)	0.07, 0.28	**3.4**

SE = standard error; CI = confidence interval; MVPA = moderate-to-vigorous physical activity.

*Note 1*: α–coefficients were estimated by regressing the potential mediators onto the physical/social environmental perceptions.

*Note 2*: β-coefficients were estimated by regressing mental health-related quality of life onto the physical/social environmental characteristics and potential mediators.

*Note 3*: αβ-coefficients represent the mediated effect.

*Note 4*: all possible mediators were positively scored (higher score = more physical activity).

*Note 5*: all analyses were adjusted for individual-level BMI, marital status, smoking status, educational level and employment status, work-related and household-related total physical activity.

### Associations between potential mediators and mental health-related quality of life (β-coefficients; Table 3)

In model 1, transport-related cycling (CI = 0.31, 1.61) and leisure-time MVPA (CI = 0.70, 1.45) were positively associated with mental health-related quality of life after taking into account neighbourhood personal safety. In model 2, transport-related walking (CI = 0.08, 0.83), transport-related cycling (CI = 0.30, 1.59), leisure-time walking (CI = 0.48, 1.22) and leisure-time MVPA (CI = 0.56, 1.30) were positively related to mental health-related quality of life after controlling for the perceived neighbourhood physical activity environment. After taking into account social support for physical activity from family/friends (model 3), transport-related walking (CI = 0.14, 0.90), transport-related cycling (CI = 0.44, 1.74), leisure-time walking (CI = 0.52, 1.28) and leisure-time MVPA (CI = 0.59, 1.36) were positively associated with mental health-related quality of life. Finally, in model 4, leisure-time walking (CI = 0.66, 1.39) and leisure-time MVPA (CI = 0.67, 1.41) were positively associated with mental health-related quality of life after taking into account social cohesion of the neighbourhood.

### Mediated effects of physical activity behaviours on the associations between physical/social environmental perceptions and mental health-related quality of life (αβ-coefficients; Table 3)

For the association between neighbourhood personal safety and mental health-related quality of life (model 1), transport-related cycling (1.5%; CI = 0.01, 0.11) and leisure-time MVPA (1.9%; CI = 0.01, 0.14) were identified as significant mediators. The multiple mediation model showed that the joint mediating effect of all physical activity behaviours was significant (CI = 0.01, 0.17) and that 2.3% of the association between neighbourhood personal safety and mental health-related quality of life was mediated by all mediators together. This proportion is lower than the sum of the proportions mediated by separate mediators because of significant correlations between some of the mediators (3 significant correlations, coefficients ranging from r = 0.17 to r = 0.32, p<0.001).

In model 2, transport-related walking (2.1%, CI = 0.01, 0.22), transport-related cycling (1.6%, CI = 0.01, 0.17), leisure-time walking (6.1%; CI = 0.16, 0.49) and leisure-time MVPA (4.9%; CI = 0.12, 0.40) mediated the association between the perceived neighbourhood physical activity environment and mental health-related quality of life. The multiple mediation model showed that the joint mediating effect of all physical activity behaviours was significant (CI = 0.38, 0.78), mediating 11.0% of the association between the perceived neighbourhood physical activity environment and mental health-related quality of life.

In model 3, transport-related walking (5.6%, CI = 0.02, 0.18), transport-related cycling (3.7%; CI = 0.01, 0.12), leisure-time walking (15.9%; CI = 0.16, 0.40) and leisure-time MVPA (17.6%, CI = 0.19, 0.44) mediated the association between social support for physical activity from family or friends and mental health-related quality of life. The multiple mediation model showed that the joint mediating effect of all physical activity behaviours was significant (CI = 0.42, 0.74), mediating 32.9% of the association between social support for physical activity and mental health-related quality of life.

In model 4, leisure-time MVPA (3.4%; CI = 0.07, 0.28) mediated the relationship between social cohesion of the neighbourhood and mental health-related quality of life.

## Discussion

The present study showed that proximal social (social support for physical activity from family or friends), distal social (neighbourhood social cohesion), as well as broader physical environmental attributes (neighbourhood personal safety and perceived neighbourhood physical activity environment) were associated with mental health-related quality of life (as a proxy for mental health) in middle-aged and older adults. These findings are consistent with previous studies in middle-aged and older adults [12,14,19,20] and emphasize the importance of including a broad range of attributes, across different levels, when examining potential correlates of mental health in middle-aged and older adults. Furthermore, the results showed that some of these direct associations were mediated by leisure-time walking, leisure-time MVPA, transport-related walking and transport-related cycling. The strongest mediating effects were found for the relationship between perceived social support for physical activity from family/friends and mental health-related quality of life: 32.9% of this association was mediated by the different types of physical activity.

There are several possible explanations for these findings. Firstly, it has been reported that the relative contribution of proximal social environmental factors (including social support for physical activity) to explain physical activity is much stronger than the contribution of distal social and physical environmental factors, both in adults and older adults [50–52]. Furthermore, previous cross-sectional and longitudinal studies identified perceived social support from family or friends as a strong and consistent determinant of mental health in adults and older adults [12,53,54]. These results confirm that the direct and indirect associations between social support from family or friends, physical activity and mental health are strong. Consequently, the strong mediating effects can be seen as a logical consequence of these strong (in)direct associations. Second, a possible methodological explanation is that self-reported leisure-time physical activity and perceived social support for physical activity from family/friends share method variance. As the questions to assess social support were very specifically related to leisure-time physical activity (e.g. how often do friends do physical activity with you), this could have led to stronger associations with physical activity compared with for instance the associations between neighbourhood environmental factors that are not specifically related to physical activity (e.g. my neighbourhood is safe from crime) and physical activity. This pattern of shared method variance has been documented in previous studies [55,56].

Some of the associations of the broader neighbourhood social and physical environmental factors with mental health-related quality of life were also mediated by physical activity. However, these mediating effects were weaker than the mediating effect of social support for physical activity from family or friends. More specifically, 11.0%, 3.4% and 2.3% respectively, of the associations of the perceived neighbourhood physical activity environment, neighbourhood personal safety and neighbourhood social cohesion with mental health-related quality of life were mediated by physical activity. Direct associations between the distal social/physical neighbourhood environmental factors and mental health-related quality of life were present, but it seems that these associations may predominantly be explained through pathways other than physical activity. A previous study showed that sense of control and friendship quality mediated the relationship between neighbourhood social cohesion and depressive symptoms in older adults [14]. In addition, general support of family or friends has been suggested as a mediator of the relationship between neighbourhood-related problems and depressive symptoms in older adults [57,58]. Moreover, Stafford et al [23] found that psychological stress acted as a mediator of the relationship between neighbourhood safety and the likelihood of being depressed. However, because these variables were not assessed in the present study, their possible mediating role could not be examined.

Furthermore, existing evidence about the importance of the neighbourhood environment (particularly perceived neighbourhood personal safety and neighbourhood aesthetics) to explain older adults’ physical activity is mixed [31,59–61].This may explain the small mediating effects of physical activity on the relationships of the neighbourhood distal social and physical environment with mental health-related quality of life. A previous study has shown that high neighbourhood walkability is associated with improved mental health in older men [20]. In addition, neighbourhood walkability is the neighbourhood environmental factor most consistently associated with physical activity in adults [31]. Thus, future studies should include neighbourhood walkability as a correlate of mental health and to examine the mediating effects of physical activity on that relationship.

Leisure-time walking and MVPA were the strongest mediators of the associations between social/physical environmental factors and mental health-related quality of life. It has been previously reported that both leisure-time and transport-related physical activities are positively related to mental health [33]. However, the beneficial effects of leisure-time physical activity have typically been stronger than those of active transportation, perhaps because leisure-time physical activity is a consciously chosen activity that may induce feelings of distraction and of sense of mastery and success derived from achieving goals [62,63]. Active transportation on the other hand, is a physical activity that is rather performed out of necessity or financial considerations which might result in feelings of stress, due to increased likelihood of exposure to stressors such as dealing with heavy traffic or road rage [62].

The present study was conducted in middle-aged and older adults (55–65 years) and not in ‘older adults’ in the conventional sense (>65 years). Consequently, one should be careful when extrapolating the present findings to the 65+ age group. As neighbourhood attachment increases and the level of mobility decreases with increasing age [14], it could be expected that the relationship between the neighbourhood social and physical environment and mental health-related quality of life, and possibly the mediating effect of physical activity, might be even stronger in the 65+ age group compared with 55–65 year old adults that were included here. However, when examining these associations in future studies, researchers should certainly take physical functioning into account, because this may be an important moderator in older age groups. Furthermore, other socio-demographic determinants of mental health like gender, age, relationship status and socio-economic position [5–10] should be further examined as potential moderators in future studies.

Furthermore, because of the cross-sectional nature of the WELL study, no causality-related conclusions can be drawn from the present findings. Although Diez Roux & Mair [26] and Ransford [25] proposed conceptual frameworks which suggest that physical activity may act as a mediator of the causal relationship between social and physical environmental attributes and mental health, reverse causality may be present in our results. It might be the case that individuals with good mental health tend to be more physically active and to have more positive perceptions of their social and physical environment. Future prospective studies are necessary in order to draw definite conclusions concerning the causality of the relationships.

A first limitation of the present study is the use of self-report measures to assess physical activity and perceptions of the social and physical environment. Although a validated questionnaire was used, several biases (e.g. over/under reporting, reporter perception bias) could be present. Future studies could utilise objective measures such as accelerometers to assess physical activity and geographic information systems to objectively assess the physical environment. Third, the average score for overall mental health-related quality of life was high (79.2 ± 18.0), indicating that the present sample of middle-aged and older adults had a good overall mental health. This might be due to the fact that adults who were not able to walk 100 metres were excluded from the study. These adults have a very poor physical health, which may induce mental health problems as well. In other studies, mean values for mental health-related quality of life assessed by the SF-36 are usually around 50 [22], so a response bias towards mentally healthy participants might have occurred in this study. Furthermore, the SF-36 scale assesses mental health-related quality of life, and not mental health per se. Although mental health-related quality of life can be seen as a proxy for mental health, this hampers comparison with other studies and other results may have been found when more objective measures of mental health (e.g. diagnostic interviews) were used as an outcome measure. Nonetheless, in large-scale population-based studies, it remains very challenging to obtain an accurate assessment of mental health [35]. Finally, although the response rate in the current study (38%) was similar to other studies using postal surveys, the relatively low response rate may have induced selection bias.

The present study also has important strengths. To our knowledge, this was the first study to examine the mediating effects of physical activity on the relationship between the social/physical environment and mental health-related quality of life in middle-aged and older adults. A further strength is the large study sample of approximately 4,000 middle-aged and older Australian adults. In addition, correlates of mental and physical health in older adults are largely understudied. As older adults are becoming an increasingly important population subgroup in developed Western societies, the present findings offer an important contribution to this research field.

## Conclusions

If longitudinal studies can confirm the present results, it may be suggested that tailored interventions to improve mental health-related quality of life in middle-aged and older adults should use a combined strategy, focusing on increasing perceived social support for physical activity from friends or family and leisure-time physical activity. Previous study findings emphasized the importance of general social support (e.g the warmth and friendliness of the surrounding environment) [12], but the present study showed that perceiving support specifically for physical activity may also be beneficial for mental health. This positive association works both direct and indirect through increased physical activity. Findings also suggest the importance of policies to promote and improve the neighbourhood physical activity environment, neighbourhood personal safety and neighbourhood social cohesion for improving mental health-related quality of life, although the mediating effect of physical activity on these associations is rather limited. Further research is needed to clarify the underlying mechanisms explaining these relationships. Nonetheless, although the mediating effect of physical activity on the relationship between the neighbourhood social and physical environment and mental health-related quality of life was small, these results are important as broader environmental changes have the opportunity to affect large populations [64]. Consequently, small effects can still have important implications for population health.

## Supporting Information

S1 Table Correlation matrix representing associations between explanatory variables, mediators and the outcome variable. * p<0.05, *** p<0.001. PA = physical activity, MVPA = moderate-to-vigorous physical activity, QOL = quality of life.
(DOCX)

## References

[1] Australian Bureau of Statistics. Available: http://www.abs.gov.au. Accessed 2014 July 15.

[2] Pan A, Sun Q, Okereke OI, Rexrode KM, Hu FB. Depression and risk of stroke morbidity and mortality: a meta-analysis and systematic review. JAMA. 2011;306: 1241–1249. 10.1001/jama.2011.1282 21934057 PMC3242806

[3] Van der Kooy K, van Hout H, Marwijk H, Marten H, Stehouwer C, Beekman A. Depression and the risk for cardiovascular diseases: systematic review and meta analysis. Int J Geriatr Psychiatry. 2007;22: 613–626. 17236251 10.1002/gps.1723

[4] Scott KM. Depression, anxiety and incident cardiometabolic diseases. Curr Opin Psychiatry. 2014;27: 289–293. 10.1097/YCO.0000000000000067 24840158

[5] Leach LS, Butterworth P, Olesen SC, Mackinnon A. Relationship quality and levels of depression and anxiety in a large population-based survey. Soc Psychiatry Psychiatr Epidem. 2013;48: 417–425. 10.1007/s00127-012-0559-9 22875222

[6] Mackinaw-Koons B, Vasey MW. Considering sex differences in anxiety and its disorders across the life span: a construct validation approach. Applied & Preventive Psychology. 2009;9: 191–209.

[7] Piccinelli M, Wilkinson G. Gender differences in depression. Critical review. Br J Psychiatry. 2000;177: 486–492. 11102321 10.1192/bjp.177.6.486

[8] Lorant V, Deliège D, Eaton W, Robert A, Philippot P, Ansseau M. Socioeconomic inequalities in depression: a meta-analysis. Am J Epidemiol. 2003;157: 98–112. 12522017 10.1093/aje/kwf182

[9] Balboa-Castillo T, Meon-Munoz LM, Graciani A, Rodriguez-Artalejo F, Guallar-Castillon P. Longitudinal association of physical activity and sedentary behavior during leisure-time with health-related quality of life in community-dwelling older adults. Health Qual Life Outcomes. 2011;9: 47 10.1186/1477-7525-9-47 21708011 PMC3142200

[10] Blazer DG, Hybels CF. Origins of depression in later life. Psychol Med. 2005;35: 1421–1252. 16168147 10.1017/S0033291705004411

[11] Henderson AS, Jorm AF. Some contributions to the epidemiology of dementia and depression. Int J Geriatr Psychiatry. 1997;12: 145–154. 9097207 10.1002/(sici)1099-1166(199702)12:2<145::aid-gps579>3.0.co;2-3

[12] Croezen S, Picavete HSJ, Haveman-Nies A, Verschuren WMM, de Groot LCPGM, van’t Veer P. Do positive or negative experiences of social support relate to current and future health? Results from the Doetinchem Cohort Study. BMC Public Health. 2012;12: 65 10.1186/1471-2458-12-65 22264236 PMC3275524

[13] Zunzunegui MV, Alvarado BE, Del Ser T, Otero A. Social networks, social integration, and social engagement determine cognitive decline in community-dwelling Spanish older adults. J Gerontol B Psychol Sci Soc Sci. 2003;58: S93–100. 12646598 10.1093/geronb/58.2.s93 PMC3833829

[14] Stafford M, McMunn A, De Vogli R. Neighbourhood social environment and depressive symptoms in mid-life and beyond. Ageing Soc. 2011;31: 893–910.

[15] Almedom AM. Social capital and mental health: an interdisciplinary review of primary evidence. Soc Sci Med. 2005;61: 943–964. 15955397 10.1016/j.socscimed.2004.12.025

[16] Modie-Moroka T. Does level of social capital predict perceived health in a community? A study of adult residents of low-income areas of Francistown, Botswana. J Health Popul Nutr. 2009;27: 462–476. 19761081 10.3329/jhpn.v27i4.3390 PMC2928095

[17] Stafford M, Gimeno D, Marmot MG. Neighbourhood characteristics and trajectories of health functioning: a multilevel prospective analysis. Eur J Public Health. 2008;18: 604–610. 10.1093/eurpub/ckn091 18948365 PMC2733764

[18] Whitley E, Gunnell D, Dorling D, Smith GD. Ecological study of social fragmentation, poverty, and suicide. Br Med J. 1999;319: 1034–1037. 10521194 10.1136/bmj.319.7216.1034 PMC28254

[19] Friedman D, Parikh NS, Giunta N, Fahs MC, Gallo WT. The influence of neighborhood factors on the quality of life of older adults attending New York senior centers: results from the Health Indicators Project. Qual Life Res. 2012;21: 123–131. 10.1007/s11136-011-9923-6 21604083

[20] Berke EM, Gottlieb LM, Moudon AV, Larson EB. Protective association between neighborhood walkability and depression in older men. Am Geriatr. 2007;55: 526–533. 17397430 10.1111/j.1532-5415.2007.01108.x

[21] Mair C, Diez Roux AV, Shen M, Shea S, Seeman T, Echeverria S, et al Cross-sectional and longitudinal associations of neighborhood cohesion and stressors with depressive symptoms in the multiethnic study of atherosclerosis. Ann Epidemiol. 2009;19: 49–57. 10.1016/j.annepidem.2008.10.002 19064189 PMC2763272

[22] Parra DC, Gomez LF, Sarmiento OL, Buchner D, Brownson R, Schmid T, et al Perceived and objective neighborhood environment attributes and health related quality of life among the elderly in Bogota, Colombia. Soc Sci Med. 2010;70: 1070–1076. 10.1016/j.socscimed.2009.12.024 20138418

[23] Stafford M, Chandola T, Marmot M. Association between fear of crime and mental health and physical functioning. Am J Public Health. 2007;97: 2076–2081. 17901443 10.2105/AJPH.2006.097154 PMC2040373

[24] Beard JR, Cerda M, Blaney S, Alhern J, Vlahov D, Galea S. Neighborhood characteristics and change in depressive symptoms among older residents of New York City. Am J Publ Health. 2009;99: 1308–1314. 10.2105/AJPH.2007.125104 PMC2696669 19008519

[25] Ransford CP. A role for amines in the antidepressant effect of exercise: a review. Med Sci Sports Exerc. 1982;4: 1–10. 10.1249/00005768-198201000-00001 6280014

[26] Diez Roux AV, Mair C. Neighborhoods and health. Ann N Y Acad Sci. 2010;1186: 125–145. 10.1111/j.1749-6632.2009.05333.x 20201871

[27] Gebel K, Bauman AE, Petticrew M. The physical environment and physical activity: a critical appraisal of review articles. Am J Prev Med. 2007;32: 361–369. 17478260 10.1016/j.amepre.2007.01.020

[28] Li F, Fisher J, Brownson RC. A multilevel analysis of change in neighborhood walking activity in older adults. J Aging Phys Act. 2005;12: 45–63. 10.1123/japa.13.2.145 15995261

[29] Sniehotta FF, Gellert P, Witham MD, Donnan PT, Crombie IK, McMurdo MET. Psychological theory in an interdisciplinary context: psychological, demographic, health-related, social, and environmental correlates of physical activity in a representative cohort of community-dwelling older adults. Int J Behav Nutr Phys Act. 2013;10: 106 10.1186/1479-5868-10-106 24011129 PMC3847689

[30] Trost SG, Owen N, Bauman AE, Sallis JF, Brown W. Correlates of adults’ participation in physical activity: review and update. Med Sci Sports Exerc. 2002;34: 1996–2001. 12471307 10.1097/00005768-200212000-00020

[31] Wendel-Vos W, Droomers M, Kremers S, Brug J, van Lenthe F. Potential environmental determinants of physical activity in adults: a systematic review. Obes Rev. 2007;8: 425–440. 17716300 10.1111/j.1467-789X.2007.00370.x

[32] Biddle SJH, Fox KR, Boutcher SH. Physical activity and psychological well-being London: Routledge 2007.

[33] Teychenne M, Ball K, Salmon J. Physical activity and the likelihood of depression in adults: A review. Prev Med. 2008;46: 397–411. 10.1016/j.ypmed.2008.01.009 18289655

[34] Teychenne M, Ball K, Salmon J. Physical activity, sedentary behavior and depression among disadvantaged women. Health Educ Res. 2010;25: 632–644. 10.1093/her/cyq008 20145009

[35] Mammen G, Faulkner G. Physical activity and the prevention of depression: a systematic review of prospective studies. Am J Prev Med. 2013;45: 649–657. 10.1016/j.amepre.2013.08.001 24139780

[36] World Health Organisation. Health topics Ageing and life course. Geneva, Switzerland World Health Organization 2006 Available: http://www.who.int/ageing/en. Accessed 2015 March 3.

[37] McNaughton SA, Crawford D, Ball L, Salmon J. Understanding determinants of nutrition, physical activity and quality of life among older adults: the Wellbeing, Eating and Exercise for a Long Life (WELL) Study. Health Qual Life Outcomes. 2012;10: 109 10.1186/1477-7525-10-109 22966959 PMC3479030

[38] Australian Institute of Health and Welfare. Available: http://www.aihw.gov.au. Accessed 2014 July 15.

[39] Australian Bureau of Statistics (2003) Census of population and housing: Socio-economic indexes for areas, Australia 2001. Catalogue no. 2039.0, Canberra: Australian Bureau of Statistics: 1–29.

[40] Dillman DA. Mail and telephone surveys: The total design method New York: Wiley 1978.

[41] Edwards R, Roberts I, Clarke M, DiGuiseppi C, Pratap S, Wentz R, et al Methods to increase response rates to postal questionnaires. Cochrane Database Syst Rev. 2007;2: MR000008 17443629 10.1002/14651858.MR000008.pub3

[42] Ware JE, Sherbourne CD. The MOS 36-item short-form health survey (SF-36). Conceptual framework and item selection. Med Care. 1992;30: 473–483. 1593914

[43] Mujahid MS, Diez Roux AV, Morenoff JD, Raghunathan T. Assessing the measurement properties of neighborhood scales: from psychometrics to ecometrics. Am J Epidemiol. 2007;165: 858–867. 17329713 10.1093/aje/kwm040

[44] Sampson RJ, Raudenbush SW, Earls F. Neighborhoods and violent crime: a multilevel study of collective efficacy. Science. 1997;277: 918–924. 9252316 10.1126/science.277.5328.918

[45] Craig CL, Marshall AL, Sjöström M, Bauman AE, Booth ML, Ainsworth BE, et al International Physical Activity Questionnaire: 12-country reliability and validity. Med Sci Sports Exerc. 2003;35: 1381–1395. 12900694 10.1249/01.MSS.0000078924.61453.FB

[46] Scarinci JC, Beech BM, Naumann W, Kovach KW, Pugh M, Fapohunda B. Depression, socioeconomic status, age and marital status in black women: a national study. Ethn Dis. 2002;12: 421–428. 12148715

[47] Keene ON. The log transformation is special. Stat Med. 1995;14: 811–819. 7644861 10.1002/sim.4780140810

[48] MacKinnon DP, Fairchild AJ, Fritz MS. Mediation analysis. Annu Rev Psychol. 2007;58: 593–614. 16968208 10.1146/annurev.psych.58.110405.085542 PMC2819368

[49] MacKinnon DP, Lockwood CM, Hoffman JM, West SG, Sheets V. A comparison of methods to test mediation and other intervening variable effects. Psychol Methods. 2002;7: 83–104. 11928892 10.1037/1082-989x.7.1.83 PMC2819363

[50] De Bourdeaudhuij I, Teixeira PJ, Cardon G, Deforche B. Environmental and psychosocial correlates of physical activity in Portuguese and Belgian adults. Public Health Nutr. 2005;8: 886–895. 16277805 10.1079/phn2005735

[51] De Greef K, Van Dyck D, Deforche B, De Bourdeaudhuij I. Physical environmental correlates of self-reported and objective physical activity in Belgian type 2 diabetes patients. Health Soc Care Community. 2011;19: 178–188. 10.1111/j.1365-2524.2010.00958.x 20880106

[52] Giles-Corti B, Donovan RJ. Relative influences of individual, social environmental, and physical environmental correlates of walking. Am J Public Health. 2003;93: 1583–1589. 12948984 10.2105/ajph.93.9.1583 PMC1448014

[53] Glass TA, Mendes de Leon CF, Bassuk SS, Berkman LF. Social engagement and depressive symptoms in late life: longitudinal findings. J Aging Health. 2006;18: 604–628. 16835392 10.1177/0898264306291017

[54] Melchior M, Berkman LF, Niedhammer I, Chea M, Goldberg M. Social relations and self-reported health: a prospective analysis of the French Gazel cohort. Soc Sci Med. 2003;56: 1817–1830. 12639598 10.1016/s0277-9536(02)00181-8

[55] Dishman RK, Darracott CR, Lambert LT. Failure to generalize determinants of self-reported physical activity to a motion sensor. Med Sci Sports Exerc. 1992;24: 904–910. 1406176

[56] Sallis JF, Taylor WC, Dowda M, Freedson PS, Pate RR. Correlates of vigorous physical activity for children in grades 1 through 12: comparing parent-reported and objectively measured physical activity. Pediatr Exerc Sci. 2002;14: 30–44.

[57] Brown SC, Mason CA, Spokane AR, Cruza-Guet MC, Lopez B, Szapocznik J. The relationship of neighborhood climate to perceived social support and mental health in older Hispanic immigrants in Miami, Florida. J Aging Health. 2009;21: 431–459. 10.1177/0898264308328976 19318605 PMC2933404

[58] Schieman S, Meersman SC. Neighborhood problems and health among older adults: received and donated social support and the sense of mastery as effect modifiers. J Gerontol B Psychol Sci Soc Sci. 2004;59: S89–97. 15014096 10.1093/geronb/59.2.s89

[59] Saelens BE, Handy SL. Built environment correlates of walking: a review. Med Sci Sports Exerc. 2008;40: S550–S566. 10.1249/MSS.0b013e31817c67a4 18562973 PMC2921187

[60] Van Cauwenberg J, De Bourdeaudhuij I, De Meester F, Van Dyck D, Salmon J, Clarys P, et al Relationship between the physical environment and physical activity in older adults: a systematic review. Health Place. 2011;17: 458–469. 10.1016/j.healthplace.2010.11.010 21257333

[61] Van Dyck D, Cardon G, Deforche B, Giles-Corti B, Sallis JF, Owen N, et al Environmental and psychosocial correlates of accelerometer-assessed and self-reported physical activity in Belgian adults. Int J Behav Med. 2011;18: 235–245. 10.1007/s12529-010-9127-4 21038103

[62] Asztalos M, Wijndaele K, De Bourdeaudhuij I, Philippaerts R, Matton L, Duvigneaud N, et al Specific associations between types of physical activity and components of mental health. J Sci Med Sport. 2009;12: 468–474. 10.1016/j.jsams.2008.06.009 18768366

[63] Greist JH, Klein MH, Eischens RR, Faris J, Gurman AS, Morgan WP. Running as treatment for depression. Compr Psychiatry. 1979;20: 41–54. 759100 10.1016/0010-440x(79)90058-0

[64] Sallis JF, Owen N, Fisher EB. Ecological models of health behaviour In: Glanz K, Rimer BK, Viswanath K. Health beahvior and health education: Theory, research and practice. San Francisco, CA: Jossey-Bass 2008 pp. 465–486.

