# Supplementary material for: Relationship of the Perceived Social and Physical Environment with Mental Health-Related Quality of Life in Middle-Aged and Older Adults: Mediating Effects of Physical Activity
Source: PLoS One. 2015 Mar 23;10(3):e0120475. doi: 10.1371/journal.pone.0120475 (PMC4370752; doi:10.1371/journal.pone.0120475)
Supplement: S1 Table — * p<0.05, *** p<0.001. PA = physical activity, MVPA = moderate-to-vigorous physical activity, QOL = quality of life. (DOCX) [file pone.0120475.s001.docx]

|  | Neighbourhood aesthetics | Neighbourhood personal safety | Neighbourhood PA environment | Social support for PA | Neighbourhood social cohesion | Walking for transport | Cycling for transport | Leisure-time walking | Leisure-time MVPA | Mental health-related QOL |
| --- | --- | --- | --- | --- | --- | --- | --- | --- | --- | --- |
| Neighbourhood aesthetics  Neighbourhood personal safety  Neighbourhood PA environment  Social support for PA  Neighbourhood social cohesion  Walking for transport  Cycling for transport  Leisure-time walking  Leisure-time MVPA  Mental health-related QOL |  | 0.41*** | 0.50***  0.30*** | 0.13***  0.10***  0.20*** | 0.41***  0.39***  0.35***  0.16*** | 0.03*  0.02  0.11***  0.14***  0.03 | 0.02  0.06***  0.08***  0.09***  0.01  0.07*** | 0.09***  0.06***  0.19***  0.24***  0.06***  0.21***  0.02 | 0.08***  0.07***  0.17***  0.25***  0.07***  0.07***  0.29***  0.22*** | 0.17***  0.20***  0.23***  0.14***  0.20***  0.06***  0.07***  0.14***  0.14*** |
